# 3D-printed patient-specific guides improve screw accuracy in upper cervical spine surgery: a comparative study

**DOI:** 10.3389/fneur.2026.1853420

**Published:** 2026-08-31

**Authors:** Guoqi Niu, Yalong Guo, Gong Zhou, Yang Gao, Weili Jiang, Hui Chen, Hu Nie, Tao Liu, Zheng Sun, Jiawei Cheng, Yi Yang, Jianzhong Bai

**Affiliations:** 1 Department of Orthopedics, The Second Affiliated Hospital of Bengbu Medical University, Bengbu, Anhui, China; 2 Department of Orthopedics, The Second Affiliated Hospital of Shandong First Medical University, Tai'an, China

**Keywords:** 3D printing technology, pedicle screw, spinal surgery, surgical guide plate, upper cervical spine diseases

## Abstract

**Objective:**

To evaluate the feasibility and clinical efficacy of patient-specific 3D-printed guide plates for pedicle screw placement in the upper cervical spine, compared with the conventional free-hand technique.

**Methods:**

A retrospective analysis was conducted on 39 patients who underwent upper cervical spine surgery between June 2009 and June 2022. Patients were divided into a guide plate group (*n* = 24) and a free-hand control group (*n* = 15). Surgical parameters (operative time, blood loss, fluoroscopy frequency), screw placement accuracy (assessed by Kawaguchi classification on postoperative CT), and clinical outcomes (VAS, JOA, and ASIA scores) were compared between groups.

**Results:**

A total of 141 pedicle screws were placed. The guide plate group achieved a significantly higher rate of perfect screw placement (81.8 vs. 62.3%, *P* = 0.009) and fewer grade 2 cortical breaches. Operative time, blood loss, and fluoroscopy exposure were significantly reduced in the guide plate group (all *P* < 0.05). Both groups showed significant postoperative improvements in all clinical scores (*P* < 0.05), with no significant intergroup differences at final follow-up.

**Conclusion:**

Patient-specific 3D-printed guide plates significantly improve screw placement accuracy and reduce operative time, blood loss, and radiation exposure in upper cervical spine surgery, while achieving clinical outcomes comparable to the free-hand technique. This method is a safe and effective alternative for upper cervical pedicle screw fixation.

## 1 Introduction

Upper cervical spine pathologies, including developmental anomalies, trauma, infection, and tumors, frequently result in spinal instability that can severely compromise neurological function and even be life-threatening (1, 2). Early surgical stabilization is crucial to restore spinal alignment and prevent further injury (3, 4). Among various posterior fixation techniques (5), pedicle screw constructs have gained increasing acceptance for the management of upper cervical disorders due to their superior biomechanical stability, high fusion rates, and reliable three-column fixation (6, 7).

However, the complex and variable anatomy of the upper cervical vertebrae, with narrow pedicles and close proximity to the vertebral artery and spinal cord, makes screw placement exceptionally challenging and risks serious neurovascular injury (8). Achieving accurate and safe pedicle screw insertion remains a central objective in upper cervical spine surgery (2, 9).

Conventional free-hand screw placement relies heavily on anatomical landmarks and intraoperative fluoroscopy (10). This technique is associated with considerable limitations: it often yields suboptimal accuracy, necessitates repeated fluoroscopic verification, and prolongs operative time, thereby increasing blood loss, infection risk, and radiation exposure (8). These drawbacks are particularly pronounced in patients with deformity or aberrant anatomy, where visual and fluoroscopic guidance is less reliable. Consequently, the free-hand method is gradually being supplemented by more precise, technology-assisted approaches.

Modern surgical aids such as robotic systems and computer-assisted navigation have been developed to improve screw placement precision (11). Nevertheless, their high cost, operational complexity, and steep learning curve restrict widespread clinical adoption. In contrast, advances in digital orthopedic modeling and additive manufacturing have made patient-specific three-dimensional (3D)-printed guide plates a promising alternative (12–14). Based on reverse-engineering principles, these individualized guides offer navigational-level accuracy while being cost-effective, easy to use, and capable of reducing operative duration, blood loss, and fluoroscopy frequency (15). Despite these advantages, reported clinical applications of 3D-printed guides in the upper cervical spine remain relatively limited.

To further evaluate the feasibility and clinical value of this technique, the present study designed and utilized individualized 3D-printed guide plates to assist pedicle screw placement in patients with upper cervical spine disorders. The clinical and radiological outcomes were compared with those of conventional free-hand insertion. The present study aims to provide evidence supporting the broader clinical application of this innovative surgical technique.

## 2 Materials and methods

### 2.1 Study design and patient population

This retrospective controlled study enrolled 39 patients who underwent upper cervical spine surgery for deformities (*n* = 19) or traumatic atlantoaxial fracture-dislocation (*n* = 20) between June 2009 and June 2022. Patient allocation was based on the clinical introduction date of the 3D-printed guide technique at our institution: patients treated between June 2009 and December 2014 received conventional free-hand screw placement (control group, *n* = 15), while patients treated from January 2015 to June 2022 received guide-assisted surgery (guide group, *n* = 24). This study was approved by the institutional ethics committee.

### 2.2 Inclusion and exclusion criteria

*Inclusion criteria:* (1) diagnosis of upper cervical deformity, fracture-dislocation, infection, or tumor requiring posterior fixation; (2) pedicle morphology suitable for screw insertion as assessed on preoperative computed tomography (CT); (3) physiological ability to tolerate surgery; (4) provision of informed consent for the use of patient-specific guides; and (5) availability of complete clinical data with a minimum one-year follow-up.

*Exclusion criteria:* (1) anatomical contraindications, such as an atlantoaxial pedicle too narrow or an anomalous vertebral artery course precluding safe screw placement; (2) poor general medical condition posing a high surgical risk; and (3) psychiatric illness impairing compliance or unwillingness to participate.

### 2.3 Guide plate design and fabrication

#### 2.3.1 3D Anatomical model reconstruction

**Figure 1:**
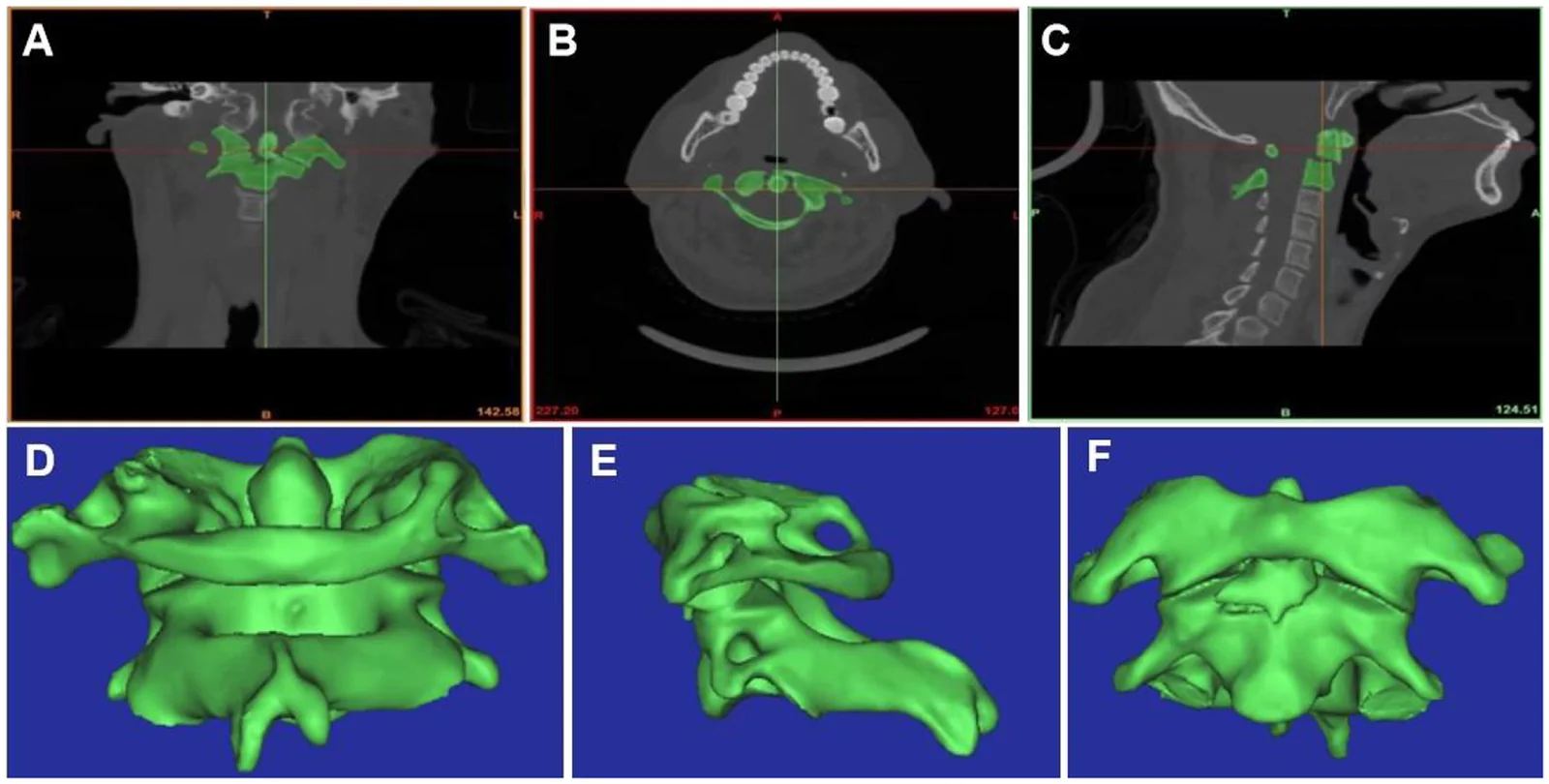
**(A-C)** CT images of the atlantoaxial vertebrae in the coronal, axial, and sagittal planes; **(D-F)** 3D reconstruction model of the atlantoaxial joint.

Preoperative thin-slice cervical CT scans (slice thickness: 0.625 mm) were obtained for patients in the guide plate group. The DICOM data were imported into Mimics 17.0 software (Materialize, Belgium). The bony structures of C1 and C2 were segmented (Figures 1A–C) to generate a 3D surface model (Figures 1D–F), which was exported in STL format.

#### 2.3.2 Virtual screw trajectory planning and guide design

**Figure 2:**
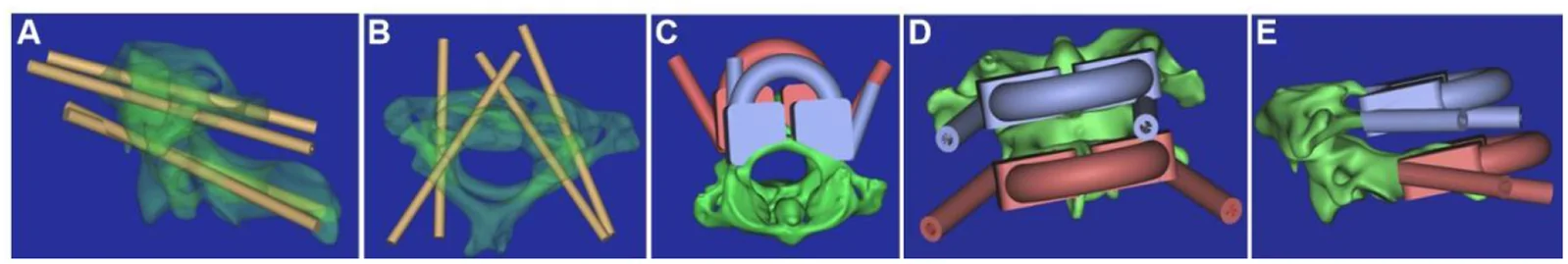
**(A)** Simulated screw trajectory from the lateral view after transparency rendering; **(B)** Simulated screw trajectory from the superior view after transparency rendering; **(C)** Superior view of the guide; **(D)** Posterior view of the guide; **(E)** Lateral view of the guide.

Within Mimics, a cylindrical model (diameter 3.5 mm) was used to simulate the pedicle screw. An experienced spine surgeon determined the optimal entry point and trajectory by visualizing the cylinder within the pedicle walls on multiplanar views (Figures 2A, B). A protective sleeve (inner diameter 2.0 mm) was designed along this trajectory. The guide plate was modeled as a semi-arched bridge structure with bilateral extensions to securely fit over the spinous process and contact the posterior elements (posterior arch, lamina, and partial facet joints) of C1 and C2. Using reverse engineering principles, a negative mold of the bone surface was created. Boolean operations merged this surface with the protective sleeve to form the integrated guide plate (Figures 2C–E). The final design was exported as an STL file.

#### 2.3.3 3D printing and preoperative validation

**Figure 3:**
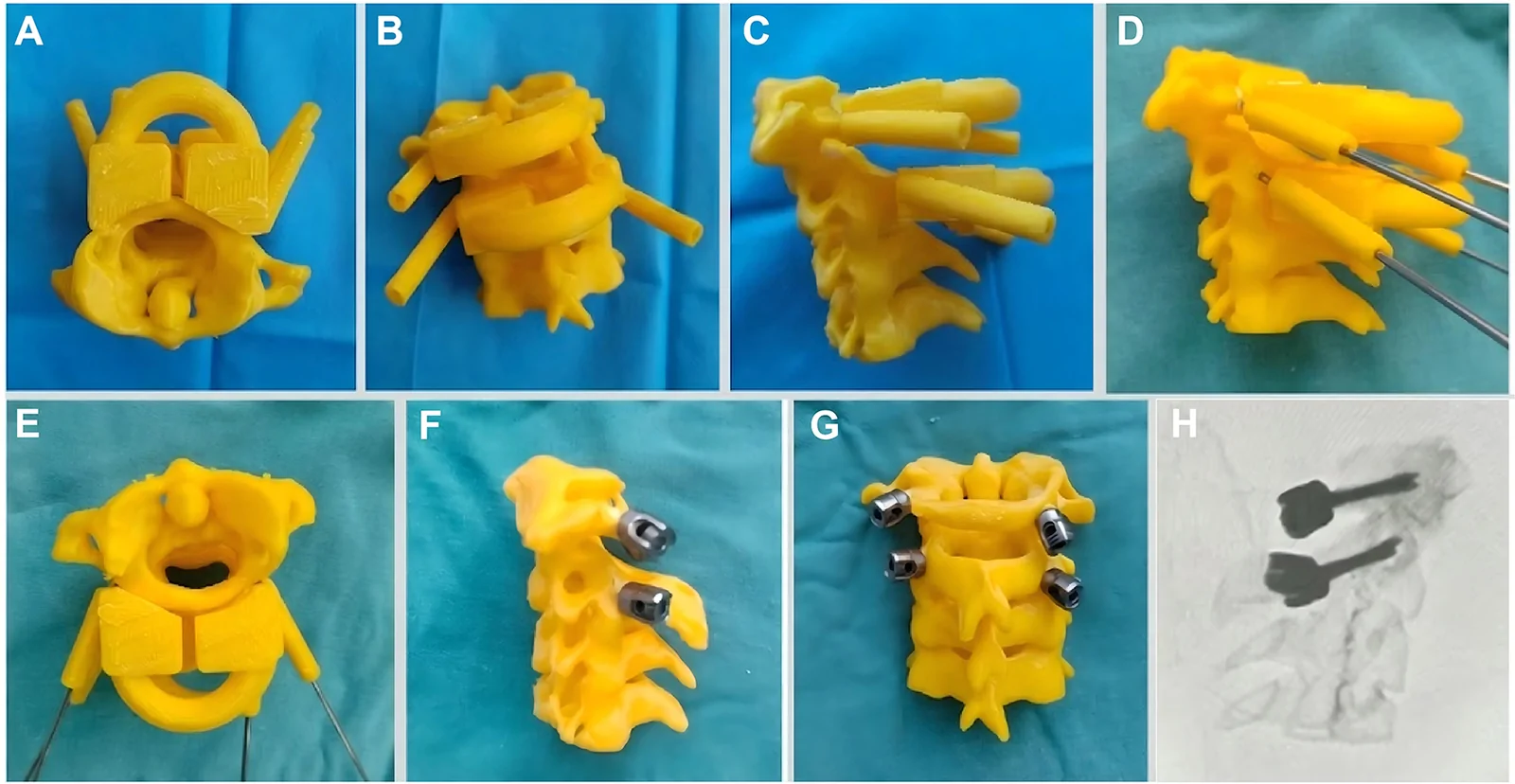
**(A)** Superior view of the guide and model; **(B)** Posterior view of the guide and model; **(C)** Lateral view of the guide and model; **(D)** Lateral view of Kirschner wire insertion along the guide sleeve; **(E)** Superior view; **(F)** Lateral view of the model after simulated screw placement *in vitro*; **(G)** Posterior view; **(H)** Intraoperative fluoroscopy.

The STL files were processed using IdeaMaker slicing software and printed on a fused deposition modeling (FDM) 3D printer with medical-grade polylactic acid (PLA) filament (layer thickness: 0.2 mm; infill density: 100%; Figures 3A–C). After printing, supports were removed and surfaces were smoothed. The fit and accuracy of the guide were verified on its corresponding 3D-printed anatomical model.

#### 2.3.4 Simulated screw placement In Vitro

The anatomical model was securely fixed, and the sterilized guide plate was positioned on its posterior surface. A 2.0 mm K-wire was drilled through the guide sleeve to the pre-planned depth under fluoroscopic guidance (Figures 3D, E). Wire position and pedicle wall integrity were confirmed visually and radiographically. The tract was then tapped, and a 3.5 mm pedicle screw was inserted (Figures 3F–H). Following successful simulation, the guide plate was sterilized for surgical use.

### 2.4 Surgical technique

#### 2.4.1 Guide plate-assisted surgery (guide group)

**Figure 4:**
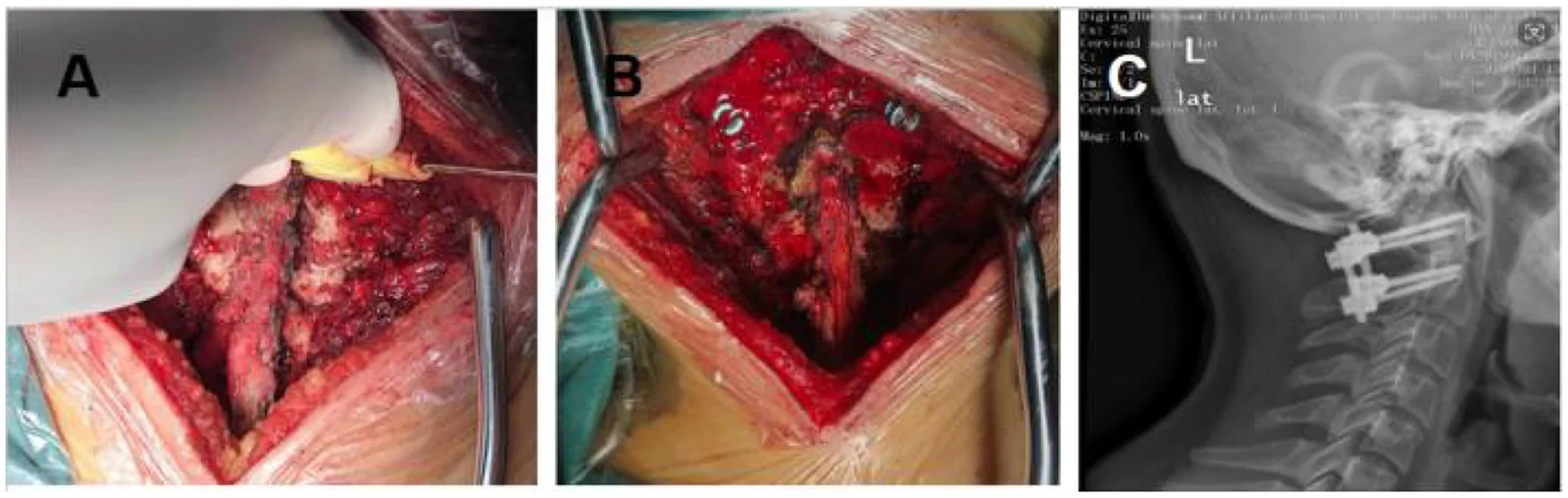
Intraoperative use of the guide for screw placement. **(A)** Drilling after the guide is fitted to the bone surface during surgery; **(B)** Intraoperative screw placement; **(C)** Postoperative X-ray review.

Under general anesthesia, patients were positioned prone with the neck slightly flexed under skull traction (3 kg). A standard posterior midline incision exposed the occiput, C1 posterior arch, C2 lamina, and lateral masses. Soft tissue was meticulously cleared from the bone surfaces at the guide's contact points. The patient-specific guide was firmly seated onto the posterior elements of C1 and C2 (Figure 4A). A 2.0 mm K-wire was drilled through the guide sleeve to the predetermined depth, and its position was verified with C-arm fluoroscopy. After removing the guide and K-wire, the pedicle tract was palpated with a ball-tipped probe, tapped, and the appropriate pedicle screw was inserted (Figure 4B). Final screw positions were confirmed fluoroscopically (Figure 4C).

#### 2.4.2 Free-hand surgery (control group)

Surgery proceeded *via* the same posterior approach. The screw entry point and trajectory were determined based on preoperative imaging and anatomical landmarks. A hand drill was advanced incrementally along the planned path, with frequent palpation of the pedicle walls using a probe to ensure integrity. The tract was then tapped, and the screw was placed.

### 2.5 Postoperative management

Postoperative care was standardized for both groups. Patients received symptomatic management including steroids, analgesics, antibiotics, and gastrointestinal protectants. Early mobilization was encouraged: patients began log-rolling on postoperative day 1, progressed to assisted ambulation with a cervical collar by day 3, and underwent postoperative radiographic evaluation (X-ray and 3D-CT) on day 4 to assess screw placement accuracy.

### 2.6 Outcome measures

Primary outcome measures included: (1) Surgical parameters: operative time (specific to the pedicle screw insertion segment), intraoperative blood loss, and fluoroscopy frequency; (2) Radiological accuracy: assessed on postoperative 3D-CT scans by two independent spine surgeons who were not involved in the index surgeries and were blinded to group allocation. Inter-observer reliability was assessed using Cohen's kappa coefficient. Screw placement was graded according to the Kawaguchi classification (16), where Grade 0 (screw entirely within pedicle) and Grade 1 (cortical breach ≤ 2 mm without complications) were defined as clinically acceptable; Grade 2 (breach >2 mm) and Grade 3 (with neurovascular complication) were defined as unacceptable; (3) Clinical outcomes: evaluated using the JOA score (17), VAS, and ASIA scale (18) preoperatively, at 1 week postoperatively, and at the final follow-up. Fusion status was assessed at the final follow-up using CT (presence of continuous trabecular bone bridging across the atlantoaxial joint) and dynamic radiographs (< 3° of angular motion on flexion-extension views). Fusion was evaluated by two independent observers blinded to group allocation.

### 2.7 Statistical analysis

Statistical analysis was performed using SPSS 27.0 and R 4.2.0. Continuous data are presented as mean ± SD. Normality was assessed using the Shapiro-Wilk test and homogeneity of variances using Levene's test. Normally distributed data were analyzed using paired or independent *t*-tests; otherwise, Wilcoxon signed-rank or Mann-Whitney U tests were used. For repeated measures (JOA, VAS, and ASIA), linear mixed-effects models with patient-level random intercepts and fixed effects for time, group, and their interaction were applied, with pairwise comparisons adjusted by Bonferroni-Holm correction. Screw-level accuracy was analyzed using mixed-effects logistic regression with random intercepts per patient to account for within-patient clustering. Categorical data were compared using the Chi-square test. *Post-hoc* power analysis for the primary outcome was performed using a two-proportion z-test with conservative correction for clustering (ICC = 0.3). Two-sided *P* < 0.05 was considered significant.

## 3 Results

### 3.1 Baseline patient characteristics

The two groups were comparable at baseline, with no statistically significant differences in demographic characteristics, diagnosis distribution, or preoperative functional scores (all *P* > 0.05). Detailed data are summarized in Table 1.

**Table 1:** Baseline characteristics and preoperative functional scores of patients.

Variable	Control (*n* = 15)	Guide (*n* = 24)	t/χ^2^	*P*-value
Age (years, Mean±SD)	40.07 ± 9.68	47.33 ± 11.82	−1.98	0.06
Gender (male/female, *n*)	8/7	15/9	0.31	0.58
follow-up duration (months)	56.2 ± 20.5	45.8 ± 18.3	1.82	0.074
Diagnosis (*n*)				
Deformity	8	11	0.20	0.65
Traumatic fracture/dislocation	7	13		
JOA score	9.07 ± 1.49	9.79 ± 1.98	−1.22	0.23
VAS score	6.20 ± 1.27	6.38 ± 1.66	−0.35	0.73
ASIA sensory score	198.67 ± 12.76	201.16 ± 13.48	−0.58	0.57
ASIA motor score	87.27 ± 8.42	89.33 ± 10.63	−0.64	0.53

### 3.2 Surgical parameters

All procedures were completed successfully by the same surgical team, with no intraoperative injuries to the vertebral artery, spinal cord, or nerve roots, and postoperative imaging confirmed satisfactory implant positioning without evidence of loosening, migration, or breakage. The guide plate group demonstrated significant advantages over the control group across all surgical parameters, including significantly shorter operative time (141.54 ± 50.09 min vs. 197.47 ± 61.12 min, *P* = 0.004), reduced intraoperative blood loss (179.83 ± 65.17 mL vs. 338.67 ± 126.55 mL, *P* < 0.001), and lower fluoroscopy frequency (13.42 ± 8.19 times vs. 29.87 ± 10.11 times, *P* < 0.001). Detailed results are presented in Table 2.

**Table 2:** Comparison of surgical indicators between the two groups of patients.

Group	Cases	Operation time (min)	Blood loss (mL)	Fluoroscopy (times)
Control	15	197.47 ± 61.12	338.67 ± 126.55	29.87 ± 10.11
Guide	24	141.54 ± 50.09	179.83 ± 65.17	13.42 ± 8.19
*t*-value	-	3.12	4.50	5.57
*P*-value	-	0.004	< 0.001	< 0.001

### 3.3 Accuracy of screw placement

**Figure 5:**
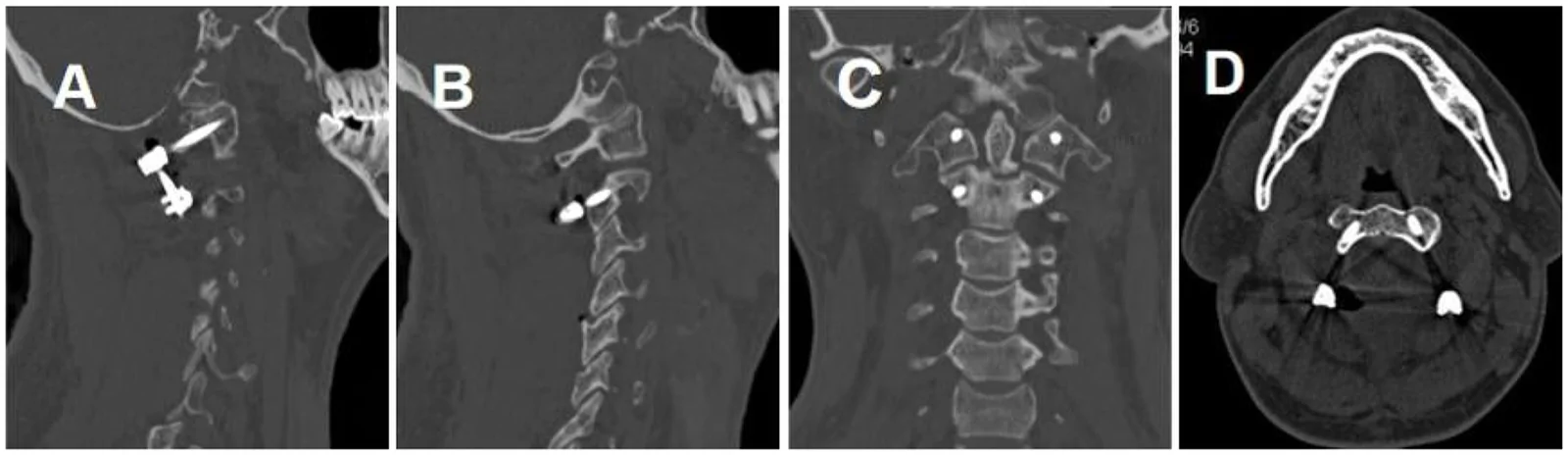
Postoperative imaging studies. **(A, B)** Sagittal, **(C)** coronal, and **(D)** transverse views of the three-dimensional computed tomography (3D-CT) reconstruction of the cervical spine.

A total of 141 pedicle screws were placed (88 in the guide plate group and 53 in the control group). Inter-observer agreement for Kawaguchi grading was substantial (κ = 0.84, 95% CI 0.76–0.91). Postoperative 3D-CT evaluation using the Kawaguchi classification (Figure 5) revealed significantly higher accuracy in the guide plate group. The rate of clinically acceptable screw placement (Grade 0 or 1) was 97.7% (86/88) in the guide plate group compared to 81.1% (43/53) in the control group (OR = 6.2, 95% CI 1.6–24.3, *P* = 0.008), with perfectly placed screws (Grade 0) accounting for 81.8% (72/88) vs. 62.3% (33/53; OR = 2.8, 95% CI 1.3–6.1, *P* = 0.009), while unacceptable placement (Grade 2 or 3) occurred in only 2.3% (2/88) of screws in the guide plate group vs. 18.9% (10/53) in the control group. Detailed accuracy data are summarized in Table 3.

**Table 3:** Accuracy of screw placement: acceptability and grading.

Group	Cases	Total	Grade 0	Grade 0,1
Control	15	53	33	43
Guide	24	88	72	86
OR (95% CI)	-	-	2.8 (1.3–6.1)	6.2 (1.6–24.3)
*P*-value^*^	-	-	0.009	0.008

^*^P values were derived from mixed-effects logistic regression.

Stratified analysis by vertebral level showed that the guide technique significantly improved accuracy at both C1 and C2 levels. For C1 screws, the acceptable placement rate was 97.2% (35/36) in the guide group vs. 76.2% (16/21) in the control group (*P* = 0.02). For C2 screws, the acceptable placement rate was 98.1% (51/52) vs. 84.4% (27/32), respectively (*P* = 0.03).

### 3.4 Postoperative clinical outcomes

Both surgical techniques led to significant and sustained improvements in all clinical outcome measures from preoperative baselines to the final follow-up (all *P* < 0.01 for within-group comparisons).

*JOA scores:* Although the guide plate group showed a marginally higher mean score at 1 week postoperatively, no statistically significant differences were observed between the two groups at any evaluation time point (preoperative, 1-week, or final follow-up; Table 4).

**Table 4:** Comparison of JOA scores between the two groups.

Group	Cases	Pre-op	1 week	Final follow-up	*t* (Pre-op vs. 1 week)	*P* (Pre-op vs. 1 week)	*t* (Pre-op vs. final)	*P* (Pre-op vs. final)
Control	15	9.07 ± 1.49	11.53 ± 2.03	15.13 ± 1.30	−5.76	< 0.001	−15.10	< 0.001
Guide	24	9.79 ± 1.98	12.96 ± 2.24	15.62 ± 1.47	−7.68	< 0.001	−14.92	< 0.001
t-value	-	−1.22	−1.92	−1.02	-	-	-	-
*P*-value	-	0.23	0.06	0.31	-	-	-	-

*P*-values were derived from linear mixed-effects models with Bonferroni-Holm correction for multiple comparisons.

*VAS scores:* Pain scores decreased significantly in both groups postoperatively. At the 1-week assessment, the guide plate group exhibited a lower mean VAS score (2.46 ± 0.88) than the control group (3.00 ± 0.76), although this difference approached but did not reach statistical significance (*P* = 0.057). By the final follow-up, both groups reported minimal pain with no significant intergroup difference (Table 5).

**Table 5:** Comparison of VAS scores between the two groups.

Group	Cases	Pre-op	1 week	Final follow-up	*t* (Pre-op vs. 1 week)	*P* (Pre-op vs. 1 week)	*t* (Pre-op vs. final)	*P* (Pre-op vs. final)
Control	15	6.20 ± 1.27	3.00 ± 0.76	0.60 ± 0.74	10.65	< 0.001	16.30	< 0.001
Guide	24	6.38 ± 1.66	2.46 ± 0.88	0.38 ± 0.65	12.55	< 0.001	14.65	< 0.001
*t*-value	-	−0.35	1.86	0.96	-	-	-	
*P*-value	-	0.73	0.06	0.34	-	-	-	

*P*-values were derived from linear mixed-effects models with Bonferroni-Holm correction for multiple comparisons.

*ASIA scores:* Both ASIA sensory and motor scores improved markedly after surgery in both cohorts. There were no significant differences between the guide plate and control groups at the preoperative, 1-week postoperative, or final follow-up assessments (Tables 6 and 7).

**Table 6:** Comparison of ASIA sensory scores between the two groups.

Group	Cases	Pre-op	1 Week	Final Follow-up	*t* (Pre-op vs. 1 week)	*P* (Pre-op vs. 1 week)	*t* (Pre-op vs. final)	*P* (Pre-op vs. final)
Control	15	198.67 ± 12.76	210.53 ± 6.92	221.07 ± 4.20	−6.02	< 0.001	−6.15	< 0.001
Guide	24	201.16 ± 13.48	213.50 ± 5.37	222.21 ± 4.03	−6.18	< 0.001	−9.35	< 0.001
*t*-value	-	−0.58	−1.43	−0.81	-	-	-	
*P*-value	-	0.57	0.16	0.42	-	-	-	

*P*-values were derived from linear mixed-effects models with Bonferroni-Holm correction for multiple comparisons.

**Table 7:** Comparison of ASIA motor scores between the two groups.

Group	Cases	Pre-op	1 week	Final follow-up	*t* (Pre-op vs. 1 week)	*P* (Pre-op vs. 1 week)	*t* (Pre-op vs. final)	*P* (Pre-op vs. final)
Control	15	87.27 ± 8.42	92.27 ± 5.64	98.87 ± 1.77	−3.60	0.003	−5.82	< 0.001
Guide	24	89.33 ± 10.63	95.00 ± 5.67	98.92 ± 1.82	−4.60	< 0.001	−4.98	< 0.001
*t*-value	-	−0.64	−1.40	−0.08	-	-	-	
*P*-value	-	0.53	0.16	0.94	-	-	-	

*P*-values were derived from linear mixed-effects models with Bonferroni-Holm correction for multiple comparisons.

### 3.5 Fusion rates

Fusion status was assessed at the final follow-up using CT and dynamic radiographs. In the guide plate group, 23 of 24 patients (95.8%) achieved solid fusion at the final follow-up, compared to 13 of 15 patients (86.7%) in the control group. The difference between groups was not statistically significant (χ^2^ = 0.342, *P* = 0.559). The single case of non-union in the guide group and two cases in the control group were managed with continued collar immobilization; no patient required revision surgery for non-union during the follow-up period.

### 3.6 Postoperative complications

No severe neurovascular complications related to screw malposition occurred in either group. Specific complications are summarized below.

*Guide Plate Group*: One patient experienced postoperative dyspnea requiring transient intensive care unit admission and intubation, with subsequent full recovery. One case of occipital pressure ulcer was noted, which healed with enhanced wound care.

*Control Group*: Two patients developed cerebrospinal fluid leakage associated with delayed wound healing; both were managed successfully with conservative measures including head elevation and localized wound care. During follow-up, some patients in both groups reported transient neck or shoulder stiffness or discomfort, which improved with guided rehabilitation.

## 4 Discussion

The atlantoaxial region presents a unique surgical challenge due to its intricate and highly variable anatomy (19, 20). Achieving accurate pedicle screw placement in this area is technically demanding, as even minor deviations risk injury to critical neurovascular structures such as the vertebral artery, spinal cord, and nerve roots (21). Beyond immediate injury, malpositioned screws can compromise biomechanical stability, potentially leading to construct failure and spinal instability. Therefore, the pursuit of a reliable and precise method for upper cervical pedicle screw insertion is of paramount clinical importance.

Posterior atlantoaxial fusion is a well-established treatment for instability. While various techniques exist (e.g., Gallie, Brooks, and transarticular screws), cervical pedicle screw fixation, popularized since Abumi's report, has become a mainstay due to its superior three-column fixation, biomechanical strength, and high fusion rates (22). However, the procedure's inherent technical difficulty and proximity to vital structures necessitate exceptional precision.

The conventional free-hand technique, reliant on anatomical landmarks and intraoperative fluoroscopy, is limited by its dependence on two-dimensional imaging to navigate complex three-dimensional anatomy. This often results in the need for repeated fluoroscopic verification and trajectory adjustments, which prolongs operative time and increases radiation exposure. Literature reports pedicle breach rates for this technique ranging from 6.7 to 12% (23, 24). In our study, the control group exhibited a breach rate of 37.74% (20/53 screws), with nearly half of these breaches (10 screws) classified as Grade 2 (>2 mm). This high rate of significant breaches underscores the limitations and potential risks associated with the free-hand method, particularly in anatomically challenging regions.

Modern technologies, such as computer-assisted navigation and robotics, address these limitations by providing real-time, three-dimensional spatial guidance, significantly enhancing accuracy and safety (25). However, their clinical adoption is often hindered by substantial costs, procedural complexity, a steep learning curve, sensitivity to intraoperative movement requiring re-registration, and concerns regarding increased radiation exposure during setup (26, 27).

Advances in digital orthopedics have positioned 3D printing technology as a compelling alternative for spine surgery, enabling the creation of patient-specific surgical guides based on meticulous preoperative virtual planning (28, 29). The present study demonstrates its significant benefits: the guide plate group achieved a markedly lower overall pedicle breach rate of 18.18% (16/88 screws) and a significantly higher proportion of perfectly placed (Grade 0) screws (81.82 vs. 62.26%) and clinically acceptable screws (97.73 vs. 81.13%) compared to the free-hand group. These findings are corroborated by existing literature, where Guo et al. (30) reported a >20% improvement in acceptable screw placement with a substantial reduction in fluoroscopy use, Niu et al. (31) documented superior accuracy (95.7 vs. 80.0%) alongside reductions in operative time, blood loss, and fluoroscopy, and Garg et al. (28) confirmed significantly higher accuracy with 3D-printed templates in complex deformities. Furthermore, the technology offers enhanced preoperative visualization and personalized planning, directly contributing to improved intraoperative accuracy (32). Importantly, while the guide plate technique demonstrably enhanced procedural safety and efficiency—reducing technical difficulty, operative time, and radiation exposure—both groups in our study ultimately achieved comparable and significant postoperative improvements in VAS, JOA, and ASIA scores. Therefore, 3D-printed guide-assisted pedicle screw placement in the upper cervical spine is a feasible and precise method that offers a valuable and cost-effective alternative to conventional free-hand techniques.

Despite the promising outcomes, this study has several limitations. First, as a single-center, non-randomized controlled study with a relatively small sample size, potential selection bias cannot be excluded. Second, the application of 3D-printed guides requires additional preoperative time and costs for imaging processing and printing, rendering it unsuitable for emergency cases. Furthermore, the accuracy of the guide depends on its precise intraoperative fit to the bone surface, which may be compromised by inadequate soft-tissue dissection or anatomical variations. Third, the long-term clinical outcomes and cost-effectiveness of this technique need to be further validated through multicenter, randomized trials with larger sample sizes. Fourth, the chronological cohort design introduces potential confounding from technical evolution and surgeon experience over time. While the same senior surgical team performed all procedures to minimize operator-related variability, the inherent limitations of a non-randomized, single-center design remain. Additionally, the dissociation between improved screw accuracy and comparable clinical outcomes may be attributed to ceiling effects, the safety-oriented nature of accuracy (rather than functional recovery), limited statistical power, and insufficient follow-up to detect late sequelae.

## 5 Conclusion

This study demonstrates that patient-specific 3D-printed guide plates effectively translate preoperative digital planning into precise intraoperative execution for upper cervical pedicle screw placement. Compared to the conventional free-hand technique, this method significantly improves screw accuracy, reduces operative time, blood loss, and radiation exposure, while achieving comparable clinical outcomes. These findings support the conclusion that 3D-printed guide plate-assisted pedicle screw fixation is a safe, reliable, and clinically valuable alternative for upper cervical spine surgery, meriting broader clinical adoption.

## Data Availability

The original contributions presented in the study are included in the article/supplementary material, further inquiries can be directed to the corresponding authors.

## References

[1] Wang XJ Liu H Wang BY Wu TK He JB Yan L . Early outcomes of bone autografting in the bilateral atlantoaxial joints applied in posterior fusion surgery. Orthop Surg. (2024) 16:559–67. doi: 10.1111/os.13997 38214016 PMC10925506

[2] Chu EC Trager RJ Tao C. Improvement of chronic neck pain after posterior atlantoaxial surgical fusion via multimodal chiropractic care: a case report. Cureus. (2023) 15:e34630. doi: 10.7759/cureus.34630 36891015 PMC9988189

[3] Groff MW Sriharan S Lee SM Maiman DJ. Partial corpectomy for cervical spondylosis. Spine (Phila Pa 1976). (2003) 28:14–20. doi: 10.1097/00007632-200301010-00005 12544948

[4] Maki S Aramomi M Matsuura Y Furuya T Ota M Iijima Y . Paravertebral foramen screw fixation for posterior cervical spine fusion: biomechanical study and description of a novel technique. J Neurosurg Spine. (2017) 27:415–20. doi: 10.3171/2016.12.SPINE16803 28498072

[5] Pham MH Bakhsheshian J Reid PC Buchanan IA Fredrickson VL Liu JC . Evaluation of C2 pedicle screw placement via the freehand technique by neurosurgical trainees. J Neurosurg Spine. (2018) 29:235–40. doi: 10.3171/2018.1.SPINE17875 29882714

[6] Wallace DJ Vardiman AB Booher GA Crawford NR Riggleman JR Greeley SL . Navigated robotic assistance improves pedicle screw accuracy in minimally invasive surgery of the lumbosacral spine: 600 pedicle screws in a single institution. Int J Med Robot. (2020) 16:e2054. doi: 10.1002/rcs.2054 31677227

[7] Müller F Roner S Liebmann F Spirig JM Fürnstahl P Farshad M . Augmented reality navigation for spinal pedicle screw instrumentation using intraoperative 3D imaging. Spine J. (2020) 20:621–8. doi: 10.1016/j.spinee.2019.10.012 31669611

[8] Azimi P Yazdanian T Benzel EC Aghaei HN Azhari S Sadeghi S . Accuracy and safety of C2 pedicle or pars screw placement: a systematic review and meta-analysis. J Orthop Surg Res. (2020) 15:272. doi: 10.1186/s13018-020-01798-0 32690035 PMC7372824

[9] Nooh A Lubov J Aoude A Aldebeyan S Jarzem P Ouellet J . Differences between manufacturers of computed tomography-based computer-assisted surgery systems do exist: a systematic literature review. Global Spine J. (2017) 7:83–94. doi: 10.1055/s-0036-1583942 28451513 PMC5400166

[10] Wang S Zhang W Sun J Wang Y Fan J Yu Y . Detection of common anatomical landmarks and vertical trajectories for freehand pedicle screw placement. Orthop Surg. (2023) 15:1541–8. doi: 10.1111/os.13729 37183354 PMC10235156

[11] Hur JW Kim JS Ryu KS Shin MH. Accuracy and safety in screw placement in the high cervical spine: retrospective analysis of O-arm-based navigation-assisted C1 lateral mass and C2 pedicle screws. Clin Spine Surg. (2019) 32:E193–e199. doi: 10.1097/BSD.0000000000000813 30829879

[12] Memon AR Li D Hu J Wang E Zhang D Chen X . The development of computer-aided patient-specific template design software for 3D printing in cranio-maxillofacial surgery. Int J Med Robot. (2021) 17:e2243. doi: 10.1002/rcs.2243 33580624

[13] La QD Revuri N Baloch A Bachhu S Ayub M Ahmed S . Pediatric Craniovertebral junction anomalies: a literature review. Cureus. (2025) 17:e87164. doi: 10.7759/cureus.87164 40755617 PMC12315784

[14] Agarwal P Arora G Panwar A Mathur V Srinivasan V Pandita D . Diverse Applications of three-dimensional printing in biomedical engineering: a review. 3D Print Addit Manuf. (2023) 10:1140–63. doi: 10.1089/3dp.2022.0281 37886418 PMC10599440

[15] Chen H Wu D Yang H Guo K. Clinical use of 3D printing guide plate in posterior lumbar pedicle screw fixation. Med Sci Monit. (2015) 21:3948–54. doi: 10.12659/MSM.895597 26681388 PMC4687948

[16] Kawaguchi Y Nakano M Yasuda T Seki S Hori T Kimura T . Development of a new technique for pedicle screw and Magerl screw insertion using a 3-dimensional image guide. Spine (Phila Pa 1976). (2012) 37:1983–8. doi: 10.1097/BRS.0b013e31825ab547 22531473

[17] Araki M Nonoshita H Kitano S Shigematsu H Tanaka M Kawasaki S . The critical cutoff point of the Zurich claudication questionnaire and the Japanese orthopaedic association score indicating locomotive syndrome in patients with lumbar spinal canal stenosis. J Orthop Sci. (2021) 26:290–4. doi: 10.1016/j.jos.2020.02.019 32253080

[18] Qi C Cao J Xia H Miao D Liu Y Guo J . Does cervical curvature affect neurological outcome after incomplete spinal cord injury without radiographic abnormality (SCIWORA): 1-year follow-up. J Orthop Surg Res. (2022) 17:361. doi: 10.1186/s13018-022-03254-7 35883148 PMC9327310

[19] Wenning KE Hoffmann MF. Does isolated atlantoaxial fusion result in better clinical outcome compared to occipitocervical fusion? J Orthop Surg Res. (2020) 15:8. doi: 10.1186/s13018-019-1525-y 31918713 PMC6953136

[20] Muir M Rhines L Demonte F Tatsui C Raza SM. Impact of radiation therapy on outcomes after spinal instrumentation for craniocervical junction malignancies. Neurospine. (2022) 19:434–40. doi: 10.14245/ns.2244034.017 35577332 PMC9260556

[21] Wei F Liu Z Liu X Jiang L Dang G Passias PG . An approach to primary tumors of the upper cervical spine with spondylectomy using a combined approach: our experience with 19 cases. Spine (Phila Pa 1976). (2018) 43:81–8. doi: 10.1097/BRS.0000000000001007 26020844

[22] Abumi K Itoh H Taneichi H Kaneda K. Transpedicular screw fixation for traumatic lesions of the middle and lower cervical spine: description of the techniques and preliminary report. J Spinal Disord. (1994) 7:19–28. doi: 10.1097/00002517-199407010-00003 8186585

[23] Richter M Cakir B Schmidt R. Cervical pedicle screws: conventional versus computer-assisted placement of cannulated screws. Spine (Phila Pa 1976). (2005) 30:2280–7. doi: 10.1097/01.brs.0000182275.31425.cd 16227890

[24] Ludwig SC Kramer DL Balderston RA Vaccaro AR Foley KF Albert TJ . Placement of pedicle screws in the human cadaveric cervical spine: comparative accuracy of three techniques. Spine (Phila Pa 1976). (2000) 25:1655–67. doi: 10.1097/00007632-200007010-00009 10870141

[25] Cho W Job AV Chen J Baek JH. A review of current clinical applications of three-dimensional printing in spine surgery. Asian Spine J. (2018) 12:171–7. doi: 10.4184/asj.2018.12.1.171 29503698 PMC5821924

[26] Kabra DA Garg DB. Current applications of 3-dimensional printing in spine surgery. J Orthop. (2023) 41:28–32. doi: 10.1016/j.jor.2023.05.009 37287587 PMC10241647

[27] Tong Y Kaplan DJ Spivak JM Bendo JA. Three-dimensional printing in spine surgery: a review of current applications. Spine J. (2020) 20:833–46. doi: 10.1016/j.spinee.2019.11.004 31731009

[28] Garg B Gupta M Singh M Kalyanasundaram D. Outcome and safety analysis of 3D-printed patient-specific pedicle screw jigs for complex spinal deformities: a comparative study. Spine J. (2019) 19:56–64. doi: 10.1016/j.spinee.2018.05.001 29730456

[29] Weidert S Andress S Linhart C Suero EM Greiner A Böcker W . 3D printing method for next-day acetabular fracture surgery using a surface filtering pipeline: feasibility and 1-year clinical results. Int J Comput Assist Radiol Surg. (2020) 15:565–75. doi: 10.1007/s11548-019-02110-0 31897965 PMC7973705

[30] Guo F Dai J Zhang J Ma Y Zhu G Shen J . Individualized 3D printing navigation template for pedicle screw fixation in upper cervical spine. PLoS One. (2017) 12:e0171509. doi: 10.1371/journal.pone.0171509 28152039 PMC5289602

[31] Niu G Cheng J Liu L Li C Zhou G Chen H . Individualized 3D printed navigation template-assisted atlantoaxial pedicle screws vs. free-hand screws for the treatment of upper cervical fractures. Front Surg. (2022) 9:932296. doi: 10.3389/fsurg.2022.932296 36225218 PMC9549244

[32] Feng S Lin J Su N Meng H Yang Y Fei Q . 3-Dimensional printing templates guiding versus free hand technique for cervical lateral mass screw fixation: a prospective study. J Clin Neurosci. (2020) 78:252–8. doi: 10.1016/j.jocn.2020.04.008 32340846

